# Update on the treatment of musculoskeletal manifestations in chikungunya fever: a guideline

**DOI:** 10.1590/0037-8682-0517-2019

**Published:** 2020-07-31

**Authors:** Carlos Alexandre Antunes de Brito, Cláudia Diniz Lopes Marques, Melissa Barreto Falcão, Rivaldo Venâncio da Cunha, Fabrice Simon, Lilian David de Azevedo Valadares, Kleber Giovanni Luz, Carlos Frederico Campelo de Albuquerque e Melo, Dalcy de Oliveira Albuquerque, Marina Coelho Moraes de Brito, Angela Luzia Branco Pinto Duarte

**Affiliations:** 1 Universidade Federal de Pernambuco, Departamento de Medicina Clínica, Recife, PE, Brasil.; 2 Universidade Estadual de Feira de Santana, Núcleo de Pesquisa e Extensão em Vigilância da Saúde, Feira de Santana, BA, Brasil.; 3 Fundação Oswaldo Cruz, Campo Grande, MS, Brasil.; 4 Unité des Virus Émergents, Aix-Marseille Uniy-IRD 190-Inserm 1207-IHU Méditerranée Infection, 13015 Marseille, France.; 5 Hospital Getúlio Vargas de Pernambuco, Recife, PE, Brasil.; 6 Universidade Federal do Rio Grande do Norte, Instituto de Medicina Tropical, Natal, RN, Brasil.; 7 Organização Pan-Americana da Saúde, Unidade Técnica de Doenças Transmissíveis e Análise de Situação de Saúde, Brasília, DF, Brasil.; 8 Ministério da Saúde, Secretaria de Vigilância em Saúde, Brasília, DF, Brasil.; 9 Universidade Federal de Pernambuco, Centro de Ciências Médicas, Recife, Pernambuco, Brasil.; 10 Universidade Federal de Pernambuco, Hospital das Clínicas, Serviço de Reumatologia, Recife, PE, Brasil.

**Keywords:** Chikungunya fever, Musculoskeletal pain, Arbovirus, Management

## Abstract

Since the emergence of the chikungunya virus in Brazil in 2014, more than 700,000 cases have been reported throughout the country, corresponding to one-third of all cases reported in the Americas. In addition to its high attack rates, resulting in hundreds of thousands of cases, the disease has high chronicity rates with persistent joint manifestations for more than 3 months, which can spread to more than half of the patients affected in the acute phase. Pain associated with musculoskeletal manifestations, often disabling, has an effect on patients’ quality of life at different stages of the disease. Currently, the challenge faced by specialists is identifying the best therapy to be instituted for symptom relief despite the limited number of published intervention studies. In 2016, a multidisciplinary group published pharmacological treatment protocols for pain in patients with chikungunya, which was incorporated into the guidelines for clinical management of the Brazilian Ministry of Health in 2017; in that same year, a consensus was published by the Brazilian Society of Rheumatology about diagnosis and treatment. After 5 years of experience with chikungunya epidemics, in 2019, specialists involved in the protocols of the Brazilian Society of Rheumatology and Brazilian Ministry of Health prepared an update with the main objective of developing flowcharts for the therapeutic approach of musculoskeletal manifestations in adult patients to enable specialists at different levels of healthcare to spread and apply this guideline in a systematic and simplified manner.

## INTRODUCTION

The chikungunya virus (CHIKV) is an arbovirus of the family *Togaviridae*, genus *Alphavirus*. The first reports of chikungunya outbreaks were described in Tanzania in 1952, and it did not reach the Americas until December 2013. However, within a 5-year period of the virus’s arrival, 2,673,671 cases were reported, with 773,000 (29%) of them occurring in Brazil^1^ ^-^ ^3^.

In 2016, the year with the highest number of reported cases since the CHIKV was noted in Brazil, 277,882 cases were reported, with 86% of notifications concentrated in the northeast region of the country. Currently, all states of the federation have reported CHIKV cases^4^ ^-^ ^5^.

The disease has a high attack rate, and a large percentage of those infected are symptomatic compared with those infected with other arboviruses. Attack rates reach 35-75% of a population in a single epidemic^2^ ^,^ ^6^. On Réunion Island, the surveillance system during the epidemic confirmed 16,050 laboratory cases and estimated a total of 244,000 cases, which corresponded to an attack rate of 35%^7^. Moreover, approximately 40-95% of CHIKV-infected individuals may present disease symptoms^2^ ^,^ ^8^.

In Brazil, there are few seroprevalence studies; this does not allow the identification of the attack rate in different regions of viral circulation, and consequently, an estimation of the percentage of susceptible individuals in new epidemic outbreaks^8^ ^,^ ^9^. A study conducted in two cities in Bahia, which was the gateway for the virus in Brazil, identified attack rates of around 50%, with 40% of these patients becoming symptomatic in the acute phase of the disease^9^.

The percentage of patients who become chronic varies among studies, with about 40-80% of cases evolving with persistence of musculoskeletal complaints^6^ ^,^ ^9^. In a recent systematic review of 38 articles, Paixão et al*.* estimated that 43% of patients had chikungunya symptoms for more than 3 months, and the persistence of symptoms after 12 months was reported in 21% of these cases. The study suggested that the prevalence of chronification may be related to the strain of the virus and that this may be higher among the genotypes of the Indian Ocean and the Asian lineage (39%)^10^.

In the seroprevalence study by Dias et al*.* conducted in Bahia, the persistence of joint symptoms was reported in 68.1% of study participants in the neighborhood of George Américo and 75% in Alto Cemitério, higher than the prevalence reported in the literature^9^.

Brazil is a country of continental dimensions, with 8 million square kilometers and about 209 million inhabitants. When we were analyzing case reports from several states in the Southeast, Midwest, and Northern Brazil, we observed small incidence rates compared to some states in the Northeast, the epicenter of the epidemic; this finding increases the probability that millions of inhabitants in the country will be susceptible in the event of new epidemics because several regions of the country have high rates of home infestation by the *Aedes* vector^4^ ^,^ ^5^.

From a clinical presentation perspective, there is an initial pattern of acute febrile illness, but musculoskeletal manifestations are responsible for the greater frequency of the symptoms of the disease, occurring in the acute phase and persisting after the regression of fever for months to even years. 

The pain and discomfort associated with joint manifestations in their different phases causes significant physical incapacity, thereby affecting the quality of life of the patients affected with the disease. Suffering related to the infection is caused by pain and mental, mood, and sleep disorders in a significant proportion of patients^11^ ^,^ ^12^.

Pain related to CHIKV, beyond intense, is unresponsive to analgesics. In a study by Andrade et al*.* involving 106 chikungunya cases, pain intensity assessed by visual analogue scale (0-10) averaged 5.8. The authors reported that many of the patients’ pain symptoms did not respond to prescribed analgesics, with only 26% experiencing pain relief greater than 70%; it was also reported that 18.9% of the cases were characterized by neuropathic pain^13^.

Despite the importance of the topic, only three guidelines worldwide systematize the treatment of musculoskeletal disease: the first was published in 2015 by a French group^14^; the second one was published in Brazil in 2016 by a multidisciplinary team^15^, and was incorporated according to the guidelines for clinical management of the Brazilian Ministry of Health (BMH) of 2017^16^. In the same year, a consensus was published by the Brazilian Society of Rheumatology (BSR)^17^.

## METHODS

After 5 years of experience with chikungunya epidemics, in 2019, specialists involved in the protocols of the BSR and BMH prepared an update with the main objective of developing flowcharts for the therapeutic approach of the musculoskeletal manifestations in adult patients to enable specialists at different levels of healthcare spread and apply this guideline in a systematic and simplified manner. 

Because of the limited number of studies on the theme and seeking the update of Brazilian guidelines, the authors prepared a narrative review^18^ ^,^ ^19^ by consulting review articles, published guidelines, and clinical trials on the treatment of patients with musculoskeletal manifestations of any kind. The flowcharts shown in this paper were developed based on the articles consulted, and mostly on the experience of the specialists with experience on the epidemy of chikungunya.

## RESULTS AND DISCUSSION

### A therapeutic approach to musculoskeletal manifestations

Data on the literature of specific therapies for chikungunya at different stages of the disease are limited. There are no high-quality randomized clinical trials evaluating the efficacy of different therapies. Moreover, few prospective studies have evaluated drug efficacy, and these studies have several limitations, including the use of different methodologies, the lack of adequate randomization in some studies, different therapeutic doses, short follow-up times, and sometimes, a small number of cases or heterogeneous comparison groups, that make comparisons difficult. There are also other case-series studies and reported experiences of pain management experienced in epidemics^14^ ^,^ ^20^ ^-^ ^29^. 

The data currently available do not allow definitive conclusions, whether favorable or not, about the efficacy of specific therapies or between different drug therapies. Currently, recommendations are mainly based on previous experience acquired with epidemics or by extrapolating specialists’ experiences with these drugs in chronic rheumatological diseases. However, with the emergence of new, high-quality scientific evidence, these recommendations can be adjusted. 

Chronologically, the clinical spectrum of the disease involves three phases: acute (up to 14 days), post-acute (15-90 days), and chronic (after 3 months). Musculoskeletal disease represents the most frequent clinical manifestation, and its management is related to these different phases. In addition, in the post-acute and chronic phases, the therapeutic approach depends on the identification of the pattern of joint manifestation, which may be predominantly non-inflammatory/musculoskeletal (mechanical) or inflammatory with arthritis and periarticular manifestations (tenosynovitis), which may also present tendonitis, enthesitis, fasciitis, capsulitis, and periostitis. 

### Acute phase

The acute phase of CHIKV is characterized by fever and intense polyarthralgia and/or arthritis. The fever is high and continuous, with several episodes a day; it is typically more intense in the first 3 days but can persist for up to 7 days. 

Arthralgia has been described in more than 90% of adult patients in the acute phase, with a polyarticular and symmetrical pattern, mainly affecting the joints of the hands, wrists, shoulders, knees, ankles, and feet. The pain can be incapacitating and makes it difficult for patients to perform daily activities such as walking, brushing their teeth, and picking up objects. Edema (periarticular and/or articular), as well as tenosynovitis and joint stiffness, is frequently observed at this stage. 

**FIGURE 1:**
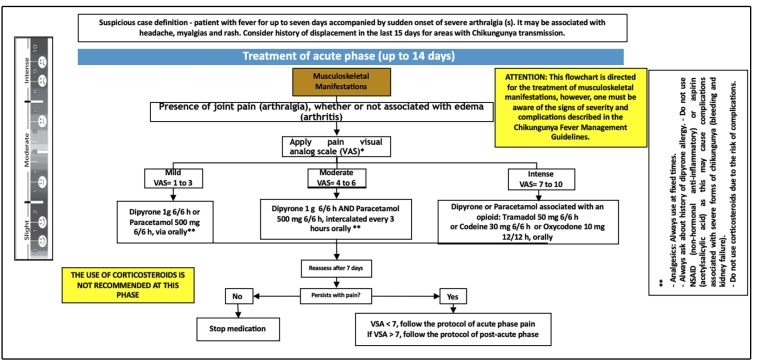
Therapeutic approach of musculoskeletal manifestations of chikungunya acute phase.

**FIGURE 2:**
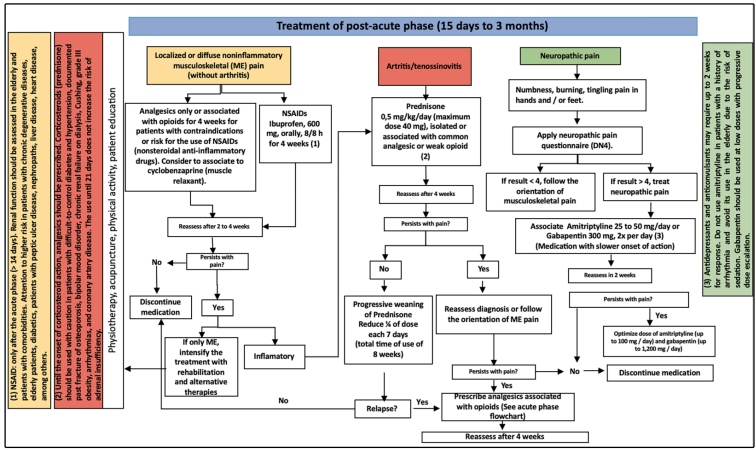
Therapeutic approach to musculoskeletal manifestations of post-acute chikungunya.

It is important for physicians to identify different clinical patterns and identify whether there is joint pain (arthralgia) associated with edema (arthritis) or not. At this stage, the treatment goal is the relief of acute pain with the use of analgesics and opioids. However, non-steroidal anti-inflammatory drugs (NSAIDs) should not be prescribed because of increased risk of bleeding or kidney damage in these patients; in addition, other acute febrile diseases may have chikungunya-like clinical manifestations in the initial phase, and NSAIDs are also contraindicated (Figure 1 and Figure 2). 

In the initial evaluation of cases with pain, it is necessary to apply the analogue visual scale of pain, thereby transforming subjective data into objective data that allow the evaluation of the therapeutic response and adequate case management. 

To avoid chronic pain, pain should be effectively treated, and the drugs used in this phase should be prescribed in fixed doses and never on an “as needed’ basis. The opioids prescribed for intense pain are potent analgesics that are safe since they are monitored, and patients are warned about any adverse events. Their main side effects are drowsiness in older people, nausea, and constipation. 

### Post-acute phase

Following the acute phase, 45-75% of adult patients have symptoms that persist after 14 days and can last up to 3 months. At this stage, fever usually disappears but musculoskeletal complaints are persistent, with polyarthralgia and/or polyarthritis of variable intensity that can range from strong to mild.

The exacerbation of pain may reach previously affected joints and may be accompanied by multiple post-acute tenosynovitis, with predominant involvement in the wrists and ankles associated with morning stiffness. Neuropathic pain and carpal tunnel syndrome are often reported. In addition, generalized symptoms such as asthenia, depression, and even alopecia may accompany this phase. 

**FIGURE 3:**
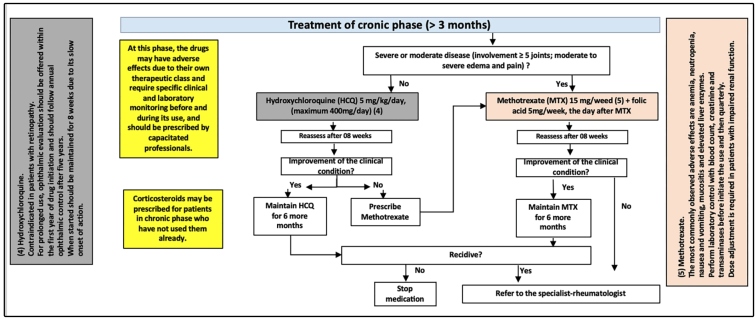
Therapeutic approach to musculoskeletal manifestations of chronic phase of chikungunya.

A clinical evaluation and eventually imaging methods are performed to define the predominant clinical pattern: a) localized or diffuse non-inflammatory musculoskeletal pain; b) arthritis/tenosynovitis (articular or periarticular disease associated with edema); and c) neuropathic pain. It is important to reinforce the possibility of a patient presenting associated pattern such as arthritis or non-inflammatory musculoskeletal pain associated with neuropathic pain (Figure 1 and Figure 3). 

Some authors suggest including other indices to evaluate the therapeutic response of the patient, such as a global evaluation by the patient and by the physician and a count of the joints with pain and edema, to calculate the index of activity and other scales commonly applied by rheumatologists. The BMH suggests a broader approach when a high number of cases are identified following an epidemic and where patients are not likely to have access to a specialist. In addition, there is no evidence that these approaches are more effective in managing cases; hence, the general practitioner must be able to manage these cases to control symptoms in the initial treatment stages. 

In the case of localized or diffuse non-inflammatory musculoskeletal pain, patients should be administered NSAIDs (ibuprofen, naproxen, celecoxib, meloxicam) and reassessed after 10 days. If the response is good, this treatment can be maintained for up to 4 weeks, with subsequent gradual reduction to withdrawal. For patients with contraindications or risks for NSAID use, analgesics isolated or associated with opioids should be prescribed for 4 weeks, with the consideration of cyclobenzaprine (a muscle relaxant). 

In disease cases with an inflammatory component (arthritis/tenosynovitis), corticosteroid use after 14 days is effective. Prednisone 0.5 milligram per kilogram per day (maximum dose being 40 milligrams) alone or in combination with a common analgesic or weak opioid should be prescribed. After 4 weeks, in patients who present a good response, the dose should be withdrawn slowly (weaning), reducing a quarter of the dose every 7 days (total time of use being 8 weeks). Abrupt suspension or rapid withdrawal may lead to a rebound of the manifestations. Contraindications to corticoid use should be considered. 

In the presence of a neuropathic component, specific therapy needs to be instituted. The Douleur Neuropathique 4, a questionnaire for neuropathic pain should be applied (Figure 1); this comprises two questions asked per interview and two questions based on physical examination for a total of 10 answers. If four or more answers are positive, the patient probably presents with neuropathic pain. In these cases, tricyclic antidepressants (amitriptyline, nortriptyline) or anticonvulsants (gabapentin, pregabalin, carbamazepine) should be used. The onset of action is slower, occurring after 2 weeks of use. In older patients, amitriptyline may lead to sedation, therefore, gabapentin is often preferred starting at low doses. Patients with a history of arrhythmia should also be administered gabapentin instead of amitriptyline. Approximately 30% of CHIKV patients may present with a neuropathic pain component associated with joint pain that fails to respond to the usual analgesics, making it necessary to associate this therapeutic class with the treatment after clinical confirmation of the condition. 

Laboratory tests should be performed before initiating drug therapy and during the use of NSAID and corticosteroid, including blood counts, fasting blood glucose, urea, creatinine, and transaminases. 

Rehabilitation with physiotherapy and alternative therapies such as acupuncture, physical activity, and patient education is important in parallel with pharmacological treatment.

### Chronic phase

Patients whose musculoskeletal complaints persist for periods longer than 3 months are considered chronic. At this stage, complaints are characterized by pain in a persistent or recurrent pattern that can maintain its intensity from previous phases, may be intense and disabling, or mild and even decreasing in intensity. In a study by Schilte et al*.* in French Polynesia, 60% of patients experienced chronification, of whom 72 were followed up for 3 years, with 45% having persistent arthralgia, 24% having initial recovery followed by recurrence, and 31% experiencing complete recovery from the acute phase^12^.

The joints most commonly affected are those of the hands, wrists, knees, and ankles, with associated morning stiffness and edema. Patients may also report the involvement of the cervical region and shoulders with determinate pain and movement limitations. 

Neuropathic pain affects approximately 20% of patients and is often neglected, which in many cases constitutes therapeutic failure because they are not responsive to analgesics and opioids, instead requiring specific therapy^12^.

The study by Schilte et al*.* analyzed the effect of the disease on the quality of life of these patients after 36 weeks and associated limitations in regard to performing their usual activities. Concerning such limitations, 62.9% of patients reported difficulty in lifting themselves up from a sitting position; 54.8% had trouble walking; 54.8% experienced difficulty picking up objects; 53.2% had trouble opening bottles and other containers; and 37.1% reported having difficulty bathing. Suffering related to CHIKV infection is not limited to pain; a significant segment of patients presented with mental, mood, and sleep disorders. The disease also had an effect on emotional status, often leading to sleep disorders (56.4%), depression (50%), memory disorders (43.5%), and concentration disorders (38.7%)^12^.

Some patients’ conditions may advance to progressive erosive arthritis with a pattern of rheumatoid or psoriatic arthritis. The authors’ experience shows that only a minor percentage of patients develop an inflammatory pattern of the chronic disease. The patients in this phase of chikungunya usually show a clinical condition of non-inflammatory pain and stiffness. However, in the evaluation of 159 patients referred to a rheumatologist, Javelle et al*.* classified 112 (70%) of them as having chronic inflammatory rheumatic disease, with 40 cases fulfilling the clinical and radiological criteria for rheumatoid arthritis, 33 for spondyloarthritis, and 21 for undifferentiated polyarthritis; none of these patients had a previous history of rheumatic disease^28^.

In different studies, several factors have been associated with the risk of chronification of musculoskeletal complaints. Factors to highlight include the following: being a female; being older than 45 years; severe pain, presence of edema, rigidity, or polyarthritis during the acute phase; previous joint disease; diabetes; high viral load; elevated levels of protein C in the acute phase; and persistently elevated immunoglobulin (Ig) M. 

**FIGURE 4:**
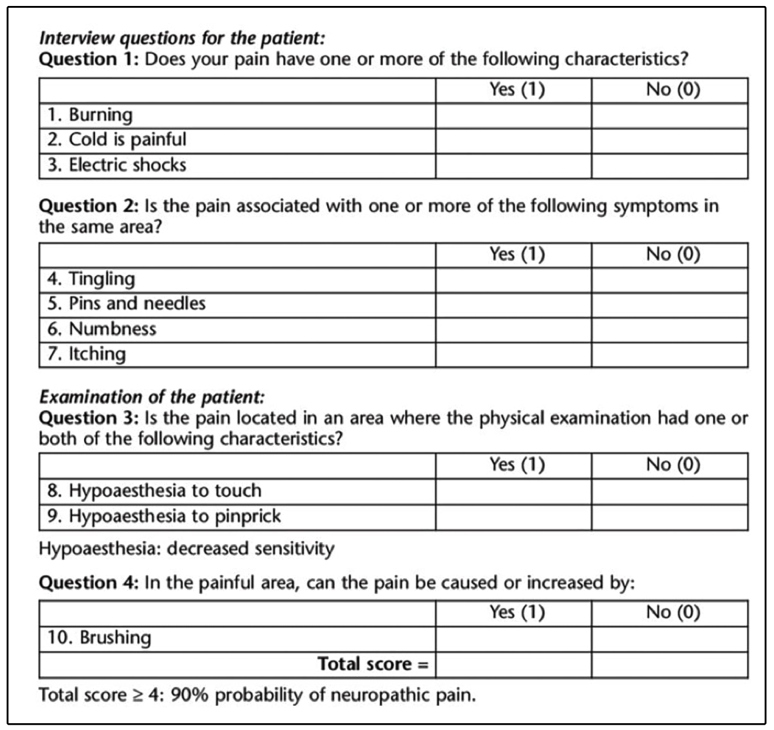
Questionnaire for diagnosis of neuropathic pain-DN4.

At this phase, analgesics, opioids, NSAIDs, and corticosteroids may be instituted for refractory or recurrent cases or as a bridge, pending the initiation of chronic-phase drugs (Figure 1 and Figure 4). Furthermore, at this stage, examinations such as simple radiography to assess joint damage may be requested. In addition, musculoskeletal ultrasonography may be useful for differentiating between joint and periarticular alterations and edema of vascular origin (useful also in the acute and post-acute phases), helping to identify other ligament or tendon injuries or erosions, and evaluating the intensity of inflammation. 

The BSR suggests that patients at this chronic stage should be tested for chikungunya IgG serology and should also have specific tests for differential diagnoses with other rheumatological diseases, including rheumatoid factor, citrullinated anti-peptide antibody, and in suspected cases of spondyloarthritis should be serchead the human leucocyte antigen B27. 

In primary care approach, or when evaluation is made by a non-rheumatology specialist, the initial evaluation may not include the performance of specific tests, leaving these to specialists following the therapeutic failure of the drugs proposed for chronic phase (Figure 1).

The drugs of choice at this stage are antimalarials, preferably hydroxychloroquine (HCQ). In this guideline, methotrexate (MTX) is an option for inflammatory joint disease (moderate or severe disease affecting more than 5 joints, moderate to severe edema and pain) and HCQ for less-severe forms. 

Patients should be evaluated after 8 weeks, and in cases of favorable clinical response, they should maintain the drug for another 6 months. In the event of failure to respond to HCQ, MTX should be prescribed. For cases of MTX failure, the physician should refer the patient to a rheumatologist. 

Most patients’ symptoms tend to decrease with the suggested therapies, leaving a small percentage of patients who require rheumatological evaluations, which will broaden the clinical investigation, consider a differential diagnosis, perform laboratory and specific imaging tests, and decide on therapeutic options to be adjusted or instituted. Some patients with chronic inflammatory joint disease who experience therapeutic failure may even require immunobiological evaluation (following recommendations used for treating rheumatoid arthritis or spondyloarthritis). 

Physicians should be aware of the adverse effects, typical of each treatment class prescribed at this stage of treatment and the need for specific clinical and laboratory monitoring before and during use. For immunosuppressive drugs, specific tests are required, such as screening for hepatitis B (hepatitis B surface antigen, antibodies to hepatitis B surface antigen, antibodies to hepatitis B core antigen), hepatitis C (antibody to hepatitis C virus) and human immunodeficiency virus, the Mantoux test, and chest x-ray. 

Physiotherapeutic treatment is recommended in all three phases of the disease, as well as other alternative therapies (acupuncture, physical activity, and patient education).

One factor that is important to acknowledge is that, in groups that have had epidemics of chikungunya, only a small percentage of patients will need drugs such as MTX. Most patients can achieve pain control with the use of NSAIDs, analgesics, and physiotherapy once the most common pattern of chronic disease seen on the authors’ clinical practice is the non-inflammatory. 

It is important to remember that this chronological division of therapy in phases is ideal, but some patients may not visit a doctor until they have reached the chronic phase (after 3 months of onset of symptoms) and thus, may never have been offered post-acute phase therapy. In this case, despite when symptoms were experienced, the previous phase therapy can be offered. 
